# Clusterin exerts a cytoprotective and antioxidant effect in human osteoarthritic cartilage

**DOI:** 10.18632/aging.103310

**Published:** 2020-06-09

**Authors:** Chiara Tarquini, Sabina Pucci, Maria Giovanna Scioli, Elena Doldo, Sara Agostinelli, Federico D’Amico, Alessandra Bielli, Amedeo Ferlosio, Emanuele Caredda, Umberto Tarantino, Augusto Orlandi

**Affiliations:** 1 Anatomic Pathology, Department of Biomedicine and Prevention, University of Rome Tor Vergata, Rome, Italy; 2 Orthopedics and Traumatology, Department of Clinical Sciences and Translational Medicine, University of Rome Tor Vergata, Rome, Italy; 3 Hygiene and Preventive Medicine, Department of Biomedicine and Prevention, University of Rome Tor Vergata, Rome, Italy; 4 Department of Biomedical Sciences, Catholic University Our Lady of Good Counsel, Tirana, Albania

**Keywords:** osteoarthritis, cartilage, clusterin, survival, oxidative stress

## Abstract

Osteoarthritis (OA) is the most common joint disease characterized by destruction of articular cartilage. OA-induced cartilage degeneration causes inflammation, oxidative stress and the hypertrophic shift of quiescent chondrocytes. Clusterin (CLU) is a ubiquitous glycoprotein implicated in many cellular processes and its upregulation has been recently reported in OA cartilage. However, the specific role of CLU in OA cartilage injury has not been investigated yet. We analyzed CLU expression in human articular cartilage *in vivo* and in cartilage-derived chondrocytes *in vitro*. CLU knockdown in OA chondrocytes was also performed and its effect on proliferation, hypertrophic phenotype, apoptosis, inflammation and oxidative stress was investigated. CLU expression was upregulated in human OA cartilage and in cultured OA cartilage-derived chondrocytes compared with control group. CLU knockdown reduced cell proliferation and increased hypertrophic phenotype as well as apoptotic death. CLU-silenced OA chondrocytes showed higher MMP13 and COL10A1 as well as greater TNF-α, Nox4 and ROS levels. Our results indicate a possible cytoprotective role of CLU in OA chondrocytes promoting cell survival by its anti-apoptotic, anti-inflammatory and antioxidant properties and counteracting the hypertrophic phenotypic shift. Further studies are needed to deepen the role of CLU in order to identify a new potential therapeutic target for OA.

## INTRODUCTION

Osteoarthritis (OA) is the most common joint disease with an increasing prevalence in the elderly population [1]. OA is a leading source of chronic pain, physical disability and reduced quality of life, resulting in a relevant clinical problem. Primary characteristics of OA are destruction of articular cartilage, subchondral bone sclerosis, osteophyte formation and synovial inflammation [2]. OA is considered a multifactorial disease and several risk factors contribute to its pathogenesis, such as aging, obesity, sex, joint misalignment, genetic predisposition and abnormal loading of the joints [3]. Chondrocytes of the articular cartilage play a central role in maintaining the dynamic equilibrium between anabolism and catabolism of the extracellular matrix under physiological conditions [4]. In OA cartilage, catabolic events prevail in the extracellular matrix [5] and quiescent chondrocytes undergo a phenotypic shift becoming hypertrophic “activated” cells, characterized by a higher proliferation rate with cluster formation and increased production of matrix-degrading enzymes, such as metalloproteinase 13 (MMP13) and aggrecanases [6]. OA-hypertrophic chondrocytes are also characterized by increased expression of terminal differentiation markers, including type X collagen (COL10A1), runt-related transcription factor 2 (RUNX2), transglutaminase 2 (TG2) and indian hedgehog (IHH) [7–9]. Several inflammatory molecular processes contribute to OA cartilage degeneration and are regulated by IL-1β, tumor necrosis factor (TNF)-α, chemokines and prostaglandins [10]. OA is also characterized by activation of oxidative stress mediators favoring the progression of the disease [11]. Inflammation, driven by cytokines, is also responsible for reactive oxygen species (ROS) generation and MMPs synthesis in OA chondrocytes [12]. NADPH oxidases (NOX) are membrane enzymes that catalyze the conversion of oxygen to superoxide. In particular, NOX4 isoform, the sole active isoform in human chondrocytes, has been reported to be upregulated in OA [12].

Despite recent advances, the mechanisms leading to cartilage consumption in patients with OA are still not fully understood and no effective therapeutic intervention exists.

Clusterin (CLU) is a ubiquitous glycoprotein constitutively expressed in many tissues and biological fluids. CLU is involved in several physiological processes, such as sperm maturation, cell-cell and cell-substrate interaction, lipid transport, membrane recycling, stress-induced chaperone activity, cell differentiation and apoptosis [13, 14]. CLU expression is increased in several age-related processes, such as neurodegenerative diseases, ischemia, but also in inflammation and cancer [15–17]. It became evident that CLU function depends on its final maturation and intracellular localization [17]. The predominant form is a secreted heterodimeric protein of ~75-80 kDa, known as secreted CLU (sCLU) [18]; sCLU is composed of disulfide-linked α and β subunits (~40 kDa) derived from an intracellular pre-secreted, glycosylated peptide (psCLU, ~60 kDa). sCLU likely acts as an extracellular molecular chaperone and exerts a cytoprotective function in pathological stress conditions as heat shock, tumor necrosis factor-α-induced cytotoxicity and UV-B irradiation [19]. A nuclear form of CLU (nCLU) has been also described, as a product of alternative splicing of pre-mRNA, and likely involved in the apoptotic response of cells to certain stimuli [14, 20]. Some authors hypothesized the involvement of CLU in OA [21, 22]. CLU is expressed in cartilage and its mRNA level has been reported to be upregulated in OA compared to healthy cartilage [21, 22]. Moreover, elevated levels of CLU protein have been found in serum and synovial fluid of patients with primary hip and knee OA [22], suggesting CLU as a potential predictive biomarker of disease progression [23]. Nevertheless, only few studies tried to define the function of increased CLU levels in OA and the exact role of CLU in articular cartilage damage and the involved cellular mechanisms remain largely unknown. Some authors reported that cultured chondrocytes supplemented with TGFβ3 produce a continuous layered articular-like cartilaginous tissue characterized by CLU accumulation, suggesting a constitutive positive role [24].

In the present work, we investigated CLU expression in human OA and non-OA cartilage and tried to define its role in the progression of the disease. We also performed experiments focused to elucidate the function of CLU in human OA cartilage-derived chondrocytes and its potential involvement in those commonly abnormal processes in OA chondrocytes, i.e. proliferation, differentiation and survival associated with altered anabolic/catabolic events and oxidative stress.

## RESULTS

### Histopathological features of OA cartilage

**Figure 1:**
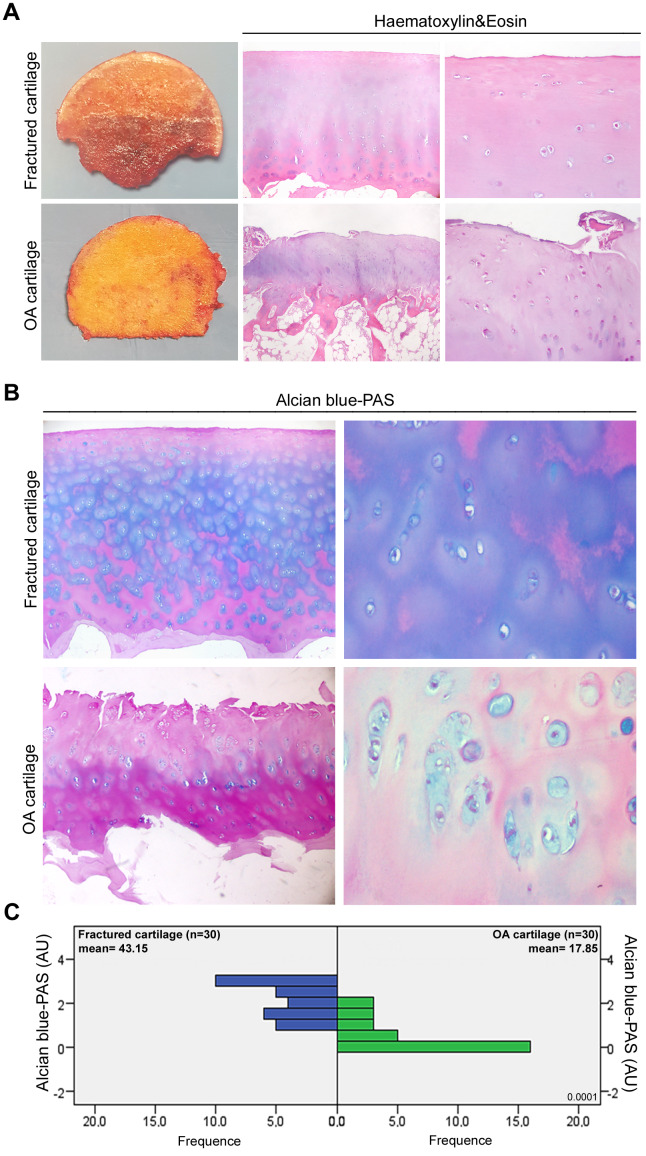
**Histopathological features of OA articular cartilage.** (**A**) Representative macroscopic images of fractured and OA femoral head samples (left panel) and Haematoxylin&Eosin-stained sections of articular cartilage at low and high magnification (center and right panel). (**B**) Representative images at different magnification of Alcian blue-PAS staining and (**C**) alcianophilia in the extracellular matrix of articular cartilage of fractured (n=30 donors; blue bars) and OA patients (n=30 donors; green bars). Abbreviations: A.U., arbitrary units. Mann-Whitney’s U-test: *P* < 0.0001.

We primarily investigated the macroscopic and microscopic features of articular cartilage. As shown in Figure 1A, articular cartilage of fractured patients appeared morphologically and microscopically normal; in contrast, OA cartilage appeared thinned and damaged, with an evident chondrocyte disorganization. OA cartilage damage was confirmed by analyzing the extracellular glycosaminoglycan accumulation (Figure 1B). In fact, Alcian blue-PAS staining in Figure 1C documented a glycosaminoglycan reduction in OA patients compared with fractured ones (*P* < 0.0001), indirectly confirming the extracellular matrix degradation in joint cartilage of OA patients. Data from each patient are reported in Supplementary Tables 1, 2.

### IL-6 and AcH4 are increased in OA cartilage

**Figure 2:**
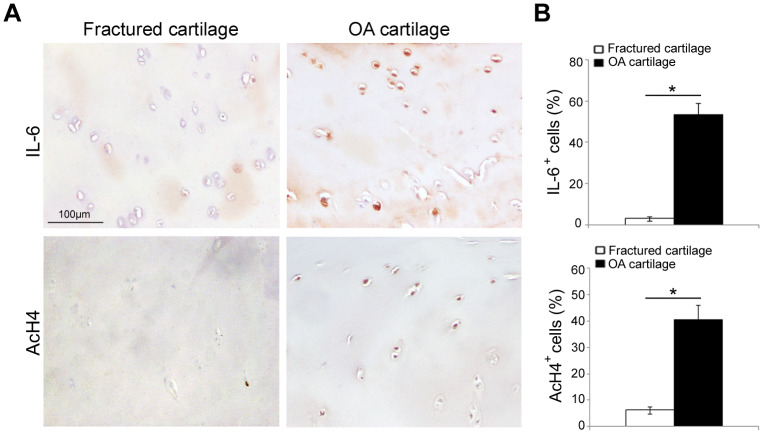
**IL-6 and AcH4 expression in articular cartilage of fractured and OA patients.** (**A**) Representative immunohistochemical images of IL-6 and AcH4 expression in femoral cartilage of fractured (n=30) and OA patients (n=30). (**B**) Bar graphs show semi-quantitative evaluation of IL-6 and AcH4. Results are expressed as mean values ± SEM. Student’s t-test: *P < 0.001.

In order to confirm the “active” inflammatory and transcriptional status of osteoarthritic cartilage [25–27], we evaluated by immunohistochemistry the expression of pro-inflammatory cytokine IL-6 and acetyl-histone H4 (AcH4), indicator of transcriptional activity, in articular cartilage of fractured and OA patients. As reported in Figure 2, IL-6 immunopositivity was greater in OA chondrocytes than in those of fractured patients (*P* < 0.001); in the latter, IL-6 expression was barely perceptible or absent. We also documented a higher percentage of AcH4 positive nuclei in OA chondrocytes compared with those of fractured patients (*P* < 0.001). Data from each patient are reported in Supplementary Tables 1, 2.

### Proliferation rate is increased in cultured human OA articular cartilage chondrocytes

**Figure 3:**
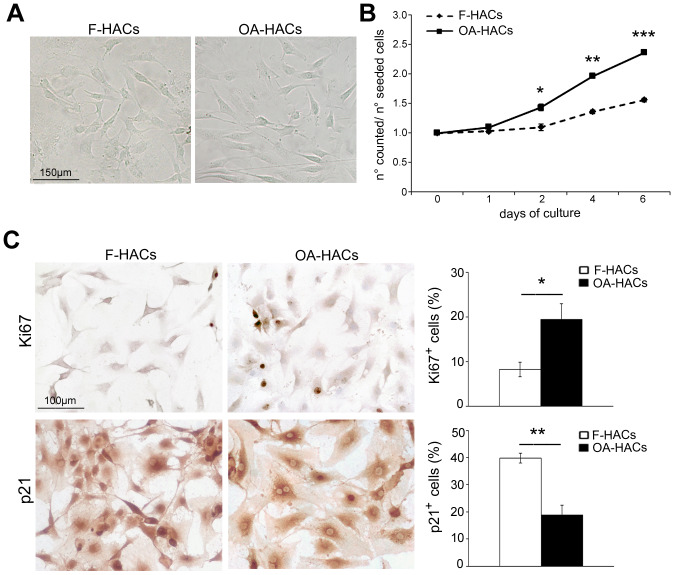
**Morphology and proliferation rate of chondrocytes from OA and fractured patients *in vitro*.** (**A**) Phase contrast micrographs of human chondrocytes in culture from articular cartilage of fractured and OA patients (F-HACs and OA-HACs). (**B**) Growth curve of F-HACs and OA-HACs in culture. (**C**) Representative images of Ki67 and p21 immunocytochemistry on F-HACs and OA-HACs. Bar graphs show the percentage of Ki67^+^ and p21^+^ cells. Data are expressed as mean values ± SEM of three independent experiments. Abbreviation: F-HACs, fractured patient human articular chondrocytes; OA-HACs, OA-Human articular chondrocytes. Student’s t-test: *P < 0.05, **P < 0.001, ***P < 0.0001.

In culture dish (Figure 3A), HACs from cartilage of fractured and OA patients exhibited a similar elongated and spindle-shaped appearance. According to the Figure 3B, although proliferation rate was generally low, OA-HACs replicated more than HACs from fractured patients, already starting from 2 days (*P* < 0.05). The increased proliferation of OA-HACs was also confirmed by immunocytochemistry. In fact, a higher percentage of Ki67^+^ nuclei was observed in OA-HACs (Figure 3C, *P* < 0.05); moreover, a lower positivity for the cell cycle inhibitor p21 was found in OA-HACs compared with HACs from fractured patients (*P* < 0.001).

### CLU is highly expressed in human OA articular cartilage chondrocytes *in vivo* and *in vitro*

**Figure 4:**
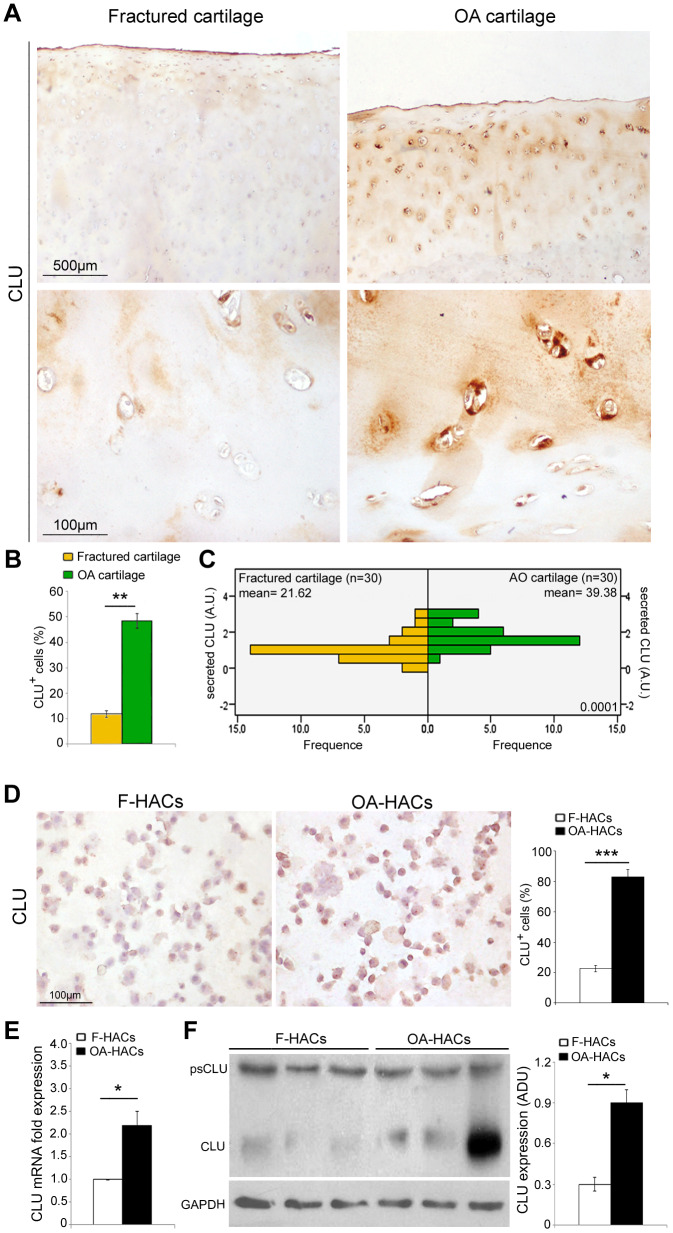
**CLU expression in cartilage and chondrocyte cultures of fractured and OA patients.** (**A**) Representative images of CLU immunohistochemistry on femoral cartilage of fractured and OA patients. (**B**) Percentage of CLU^+^ cells and (**C**) extracellular CLU secretion in femoral cartilage of fractured (n=30 donors; yellow bars) and OA patients (n=30 donors; green bars). Student’s t-test: **P < 0.001 and Mann-Whitney’s U-test: *P* < 0.0001, respectively. (**D**) Representative images and semi-quantitative evaluation of CLU immunocytochemistry on cytospinned HACs from fractured and OA patients. Data are expressed as mean values ± SEM of three independent experiments (each using pooled samples from n=3 fractured and n=3 OA donors) performed in triplicate. (**E**) CLU mRNA levels by Real-Time PCR and (**F**) representative blot and densitometric analysis of CLU protein in cultured HACs from fractured and OA cartilage of individual donors (n=10 fractured and n=10 OA patients). Experiments were performed in triplicate. Data are expressed as mean values ± SEM. Abbreviations: A.U, arbitrary units; F-HACs, fractured patient human articular chondrocytes; OA-HACs, OA-Human articular chondrocytes; psCLU, CLU precursor protein. Student’s t-test: *P < 0.01, ***P < 0.0001.

The involvement of CLU in joint degenerative diseases has been recently hypothesized. Based on that finding, we evaluated the expression of CLU in articular cartilage of OA and fractured patients. Immunohistochemical analysis (Figure 4A, 4B) documented an increased percentage of CLU^+^ cells in OA compared with fractured patients (*P* < 0.001). In addition, as shown in Figure 4C, the extracellular secretion of CLU was greater in OA cartilage compared with that of fractured patients (*P* < 0.0001). The extracellular distribution of CLU was uniform and diffuse in the intermediate-upper zone in OA cartilage, whereas in fractured patients it was limited mainly to the more superficial layers. A pericellular localization was evident, although a clear distinction between nuclear and cytoplasmic CLU staining was not appreciable. Cultured HACs from cartilage of OA and fractured patients displayed a similar result. Increased CLU expression in OA-HACs was documented by immunocytochemistry (Figure 4D; *P* < 0.0001) as well as by Real-Time PCR and Western blot that revealed higher CLU mRNA and protein levels, with some variability in the amount of CLU inside OA group (Figure 4E, 4F; *P* < 0.01).

### Clusterin silencing influences proliferation, survival, apoptosis, differentiation and catabolism of OA articular cartilage chondrocytes

**Figure 5:**
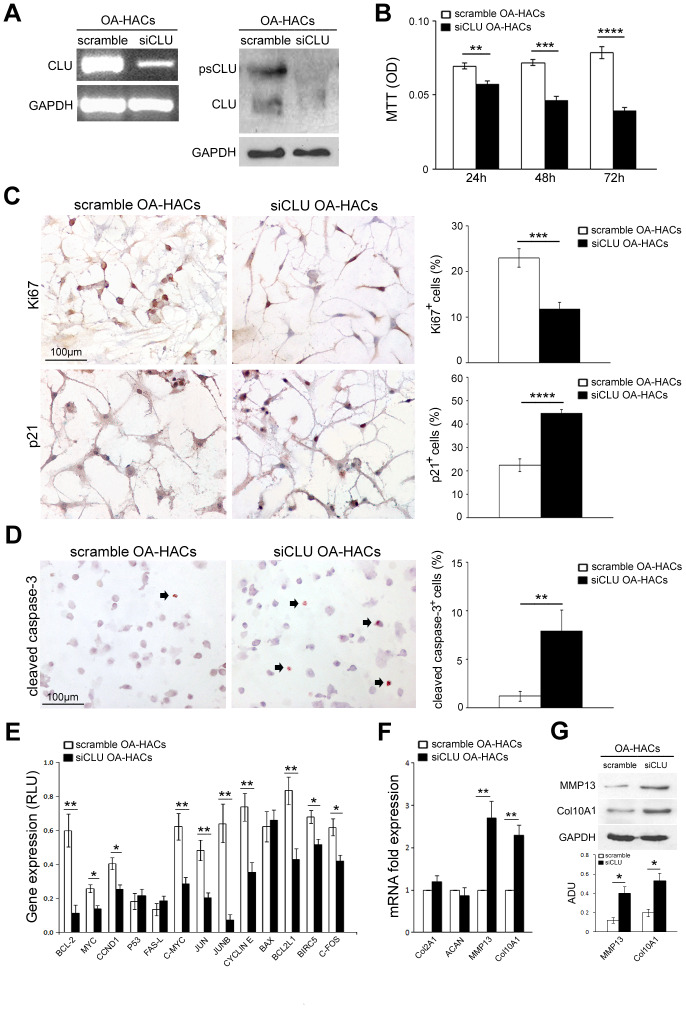
**Effects of CLU silencing on viability, proliferation/death and differentiation of cultured OA chondrocytes.** (**A**) CLU mRNA and protein expression after 48 and 72 hours, respectively, in scramble (control) and siCLU transfected OA-HACs. (**B**) Cell viability by MTT assay after 24, 48 and 72 hours of scramble and siCLU transfected OA-HACs. (**C**) Representative immunocytochemical images and bar graphs showing Ki67^+^ and p21^+^ nuclei in cultured OA-HACs and (**D**) cleaved caspase-3^+^ cytospinned OA-HACs after 72 hours of silencing. (**E**) Gene array shows the modulation of NFkB and Stat-3-related genes in siCLU transfected OA-HACs compared with scramble after 48 hours of silencing. Transcript levels were normalized on GAPDH (internal reference gene). (**F**) mRNA levels by Real-Time PCR of COL2A1, ACAN, MMP13, COL10A1, and (**G**) representative blot and densitometric analysis of MMP13 and COL10A1 in scramble and CLU-silenced OA-HACs. Data are expressed as mean values ± SEM of three independent experiments (each using pooled samples from n=3 OA donors) performed in triplicate. Abbreviations: OA-HACs, OA-Human articular chondrocytes; OD, optical density unit; RLU, relative light unit. Student’s t-test: *P < 0.05, **P < 0.01, ***P < 0.001, ****P < 0.0001.

Based on the increased expression of CLU in OA-HACs, we investigated the effect of CLU gene silencing by siRNA transfection. As shown in Figure 5A, treatment with siCLU induced a knockdown of cellular CLU mRNA and protein levels in OA-HACs; conversely, no change in CLU expression was observed in the presence of control siRNA (scramble treatment). As reported in Figure 5B, CLU silencing reduced the viability of OA-HACs compared with scramble-transfected ones, already after 24 h (*P* < 0.01), and more markedly after 48 and 72 h of treatment (*P* < 0.001 and *P* < 0.0001, respectively). Immunocytochemical analysis (Figure 5C) showed a reduced Ki67^+^ and an increased p21^+^ percentage of cultured OA-HACs after CLU silencing compared to scramble after 72h (*P* < 0.001 and *P* < 0.0001, respectively). CLU-silenced OA-HACs showed an increase of cleaved caspase-3 positive cells (Figure 5D; *P* < 0.01). Altogether, these results suggested that CLU reduction by silencing decreases proliferation and increases cell death of OA-HACs.

We also analyzed the effects of CLU silencing on NFkB and Stat-3-related gene pathways in OA-HACs. As shown in Figure 5E, cell proliferation and survival-related genes, such as BCL-2, MYC, CCND1, c-MYC, JUN, JUNB, CYCLIN E, BCL2L1, BIRC5 and C-FOS were downregulated in CLU-silenced OA-HACs compared to scramble control. During OA, HACs lose their stable (quiescent) phenotype and shift towards hypertrophic and activated one (terminal differentiation). To understand whether CLU is involved in chondrocyte differentiation and OA cartilage degradation, we analyzed the expression of COL2A1, ACAN, MMP13 and COL10A1 genes in silenced OA-HACs. As reported in Figure 5F, 5G, we documented increased MMP13 and COL10A1 transcript and protein levels in CLU-silenced OA-HACs compared with scramble (*P* < 0.01 and *P* < 0.05, respectively), whereas no changes in COL2A1 and ACAN mRNA levels were observed.

### Clusterin silencing in human OA chondrocytes increases expression of inflammation and oxidative stress markers

**Figure 6:**
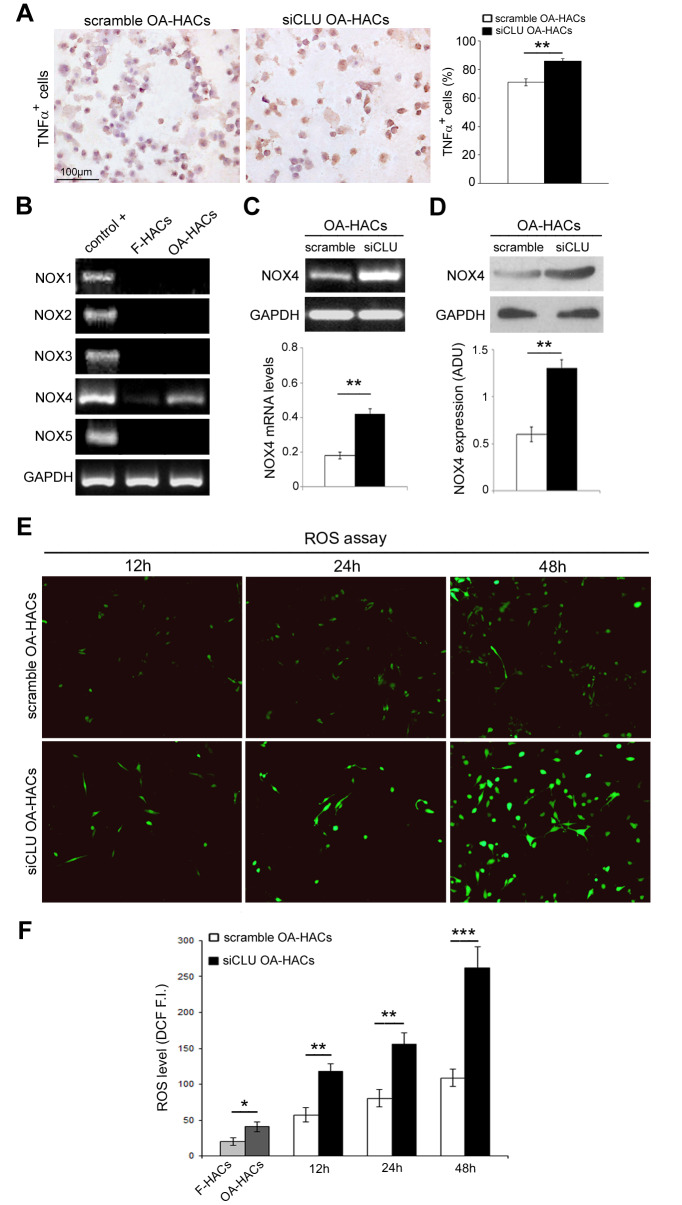
**Effects of CLU silencing on TNFα and ROS levels in cultured OA chondrocytes.** (**A**) Representative immunocytochemical images and evaluation of TNFα^+^ cytospinned OA-HACs after 72 hours of CLU silencing. (**B**) Basal mRNA expression of NOX isoforms by RT-PCR in cultured chondrocytes. (**C**, **D**) RT-PCR and blot analysis of NOX4 expression in scramble and CLU-silenced OA-HACs. (**E**, **F**) Representative images and quantitative measurement of ROS levels (green fluorescence) after 12, 24 and 48 hours of CLU silencing compared with control (scramble) and HACs from fractured patients; original magnification, 100X. Data are expressed as mean values ± SEM of three independent experiments (each using pooled samples from n=3 donors) performed in triplicate. Abbreviations: F-HACs, fractured patient human articular chondrocytes; OA-HACs, OA-Human articular chondrocytes; ADU, arbitrary densitometric unit; F.I., fluorescence intensity. Student’s t-test: *P < 0.05, **P < 0.01 and ***P < 0.001.

Inflammatory state and oxidative stress contribute to the degradation of articular cartilage during OA. In order to assess a possible role of CLU in these pathological processes, we investigated the expression of the pro-inflammatory cytokine TNFα, a direct mediator of the inflammation and oxidative stress in osteoarthritic chondrocytes [11], following CLU silencing. As shown in Figure 6A, gene silencing by CLU siRNA induced an increase in TNFα expression in OA-HACs compared with scramble control (*P* < 0.01).

Next, we analyzed the presence of NADPH oxidase (NOX) isoforms in cultured HACs, confirming NOX4 as the only isoform in human chondrocytes [11]. As reported in Figure 6B, higher NOX4 transcriptional levels were documented in OA-HACs compared to F-HACs. Then, we analyzed the effect of CLU silencing on NOX4 expression. As reported in Figure 6C, 6D, Nox4 mRNA and protein levels in CLU-silenced OA-HACs were greater than in scramble (*P* < 0.01). We also assessed the effect of CLU silencing on ROS accumulation. CLU silencing in OA-HACs induced a stronger fluorescence signal compared with scramble already after 12 h and more markedly after 48 h (Figure 6E, 6F; *P* < 0.001). In addition, as reported in Figure 6F, ROS levels were increased in OA-HACs compared with F-HACs (*P* < 0.05).

## DISCUSSION

Several inflammatory processes are activated and contribute to the degeneration of the articular cartilage during OA. In addition, oxidative stress has been reported to accelerate the progression of the disease [10–12]. Higher IL-6 expression and increased AcH4 nuclear positivity were found in OA-HACs compared with HACs from fractured patients. These findings were associated with an increased proliferation rate, a stronger Ki67 positivity and a reduced expression of the cell cycle inhibitor p21 in cultured OA-HACs. Increased IL-6 and AcH4 expression indicates an “active” inflammatory and transcriptional status of OA cartilage, also characterized by increased TNF-α expression [25–27]. Moreover, IL-6 expression was found to be related to CLU production in cancer cells and neoplastic tissues [28]. It has been described that chronic inflammation, oxidative stress, and other stress-inducing agents induce the synthesis and expression of CLU [29]. CLU is a secreted glycoprotein that seems to act as molecular chaperone with a cytoprotective function. In fact, CLU stabilizes damaged and misfolded proteins, facilitating their renaturation and/or degradation and promoting cell survival [19, 30, 31]. We confirmed the increased expression of CLU in OA *in vivo* and *in vitro*, according to literature [21, 22].

Although the overall CLU expression was higher in OA chondrocytes than in those of fractured subjects, some variability in the amount of CLU was found among OA patients. It has been reported that variability in CLU expression could indicate different states of chondrogenic differentiation as well as different stage/severity of OA [21]. Based on this evidence, we wondered whether CLU upregulation could represent an adaptive and cytoprotective response to stress condition or whether it is a mere consequence of the pathogenetic mechanisms of OA. In this regard, we silenced CLU in OA chondrocytes in order to investigate the functional role of its upregulation. CLU knockdown in OA-HACs caused a reduction of proliferation and viability as well as the concomitant increase in p21 expression. Moreover, CLU silencing downregulated genes belonging to cell proliferation signaling pathways such as BCL-2, MYC, CCND1, C-MYC, JUN, JUNB, CYCLIN E, BCL2L1, BIRC5 and C-FOS. CLU silencing also caused an increased expression of activated caspase 3, an effector enzyme of the apoptotic cascade leading to cell death [14, 32]. CLU knockdown therefore reduced survival and increased apoptosis, suggesting that CLU overexpression in OA chondrocytes is likely an adaptive response to stress condition. Our findings confirm previous observations regarding CLU involvement in growth and survival of non-tumoral and tumoral cells [14, 17, 32, 33], as shown in CLU knockout and knockdown models [34–37].

We also documented an increased TNFα expression, a direct mediator of inflammation and oxidative stress in OA chondrocytes [11], in CLU-silenced OA-HACs. The interplay between CLU and TNFα in the inflammatory process was found investigating the cytoprotective effects of CLU on TNFα-induced apoptosis of vascular smooth muscle cells and endothelial cells [38]. Moreover, it has been reported in rheumatoid arthritis synovial macrophages that the late (chronic) phase of TNFα signaling is characterized by the expression of some genes not induced during the early and acute pro-inflammatory phase [39]. CLU belongs to these late genes that are negative regulators of inflammation and NFkB signaling [39, 40] and inducers of a TNFα-mediated negative feedback loop [39]. In fact, the expression of TNFα was increased in CLU-silenced OA-HACs, sustaining the hypothesis of a possible negative feedback loop to limit excessive inflammation.

We also found an increased Nox4 expression as well as ROS accumulation in OA-HACs, as described in human articular chondrocytes under pro-inflammatory stimuli [12]. As reported above, CLU exhibits properties similar to small heat shock proteins preventing aggregation of unfolded proteins and facilitating their renaturation and/or degradation. It is a pro-survival cell factor [19, 30, 31] and protects cells from oxidative stress acting as a sort of “cellular biosensor of oxidative stress” [41]. It’s well-known that CLU silencing in human chondrocytes mimics the reduction of CLU by aging, in which OA degenerative process occurs frequently [42].

During OA progression, articular chondrocytes shift from a differentiated stable phenotype, characterized by the expression of genes such as COL2A1 and ACAN, to a hypertrophic state, characterized by terminal differentiation and the expression of COL10A1 and MMP13 [7, 43]. CLU silencing in OA-HACs induced an increased MMP13 and COL10A1 expression compared with control, supporting the hypothesis that CLU expression counteracts the phenotypic shift of OA chondrocytes. A similar pathophysiological compensatory effect of CLU upregulation has been documented in glomerular mesangial cells after injury [44]. In our model, CLU silencing mimics the terminal differentiation phase (hypertrophic state) of OA chondrocytes that fail to repair, lose the transcriptional activation status (CLU downregulation) and undergo apoptosis.

In conclusion, these data suggest a possible cytoprotective function of CLU whose upregulation counteracted hypertrophic differentiation and proliferative arrest, inflammation, oxidative stress and cell death in OA chondrocytes. Further studies are needed to define the role of CLU in order to propose it as a new therapeutic target for OA disease.

## MATERIALS AND METHODS

### Patients

In this study, we used 60 anonymized human femoral head samples, obtained from patients who underwent hip surgery in the Orthopedic Department of “Tor Vergata” University Hospital, Rome. Thirty patients (mean age 75.0 ± 1.1) underwent hip arthroplasty for primary OA and 30 patients (mean age 78.9 ± 1.6) underwent hip arthroplasty for femoral neck fracture. Before surgery, patients underwent to dual energy X-ray absorptiometry (DEXA) examination of lumbar spine and femoral neck to estimate the bone mineral density (BMD) (WHO 1994) [45]. According to the World Health Organization criteria (WHO 1994), the interpretation of the T-score is as follows: T-score> -1: normal, T-score between -1 and -2.5: osteopenia, T-score <-2.5: osteoporosis. The BMD (T-score) average values for fractured and OA patients were -2.5±0.2 and -0.3±0.3, respectively. Hip X-ray examination was performed to confirm the presence of radiological signs of OA and Kellgren–Lawrence scale was used to determine the severity of the disease [46]. Enrolled OA patients had a Kellgren-Lawrence score ≥ 3. The Harris Hip Score (HHS) was also calculated in OA patients [47], with an average value of 44.7 ± 2.6. Fracture patients had no known history of joint disease and cartilage was free of lesions (by clinical examination and X-ray) [48, 49]. Clinical-pathological features are summarized in Supplementary Tables 1, 2. The study was approved by Tor Vergata Ethical Committee and performed under written consent.

### Microscopic examination

Formalin-fixed, decalcified, paraffin-embedded femoral head samples were obtained and 5 μm-thick serial sections were cut and stained with Haematoxylin&Eosin for microscopic examination under light microscopy. Alcian blue-PAS staining was also performed to quantify glycosaminogycan accumulation in the extracellular matrix using a gradient system in arbitrary units (AU), as reported [50].

### Immunohistochemical study

For immunohistochemistry, paraffin-embedded sections (5μm-thick) were deparaffinized and the endogenous peroxidase activity blocked. Non-specific antibody binding was blocked by incubating slides with normal goat serum (Ylem, Avezzano, Italy, 1:20), then sections were incubated with mouse monoclonal anti-IL-6 (R&D System, Inc., USA, 1:20), rabbit polyclonal anti-AcH4 (EMD Millipore, USA, 1:75) and goat polyclonal anti-Clusterin β (Santa Cruz Biotechnology, USA, 1:100), with positive and negative controls. Diaminobenzidine was used as final chromogen and Mayer’s Haematoxylin as nuclear counterstain. IL-6, AcH4 and CLU immunostainings were evaluated as the percentage of positive cells in at least ten randomly selected fields at 400X magnification. Immunostaining of secreted CLU in the extracellular matrix was semi-quantitatively evaluated by using a grading system in arbitrary units as follows: absent staining (0), weak staining (1), moderate staining (2) and strong staining (3). Each observation was performed by two researchers, with an inter-observer reproducibility > 95%.

### Human chondrocyte culture and growth assay

We isolated human articular chondrocytes (HACs) from tissue samples of macroscopically OA regions (from OA patients, OA-HACs) or normal cartilage (from fractured patients, F-HACs), respectively (see “Patients” paragraph). Small cartilage samples were finely minced within 1 hour after surgery. For enzymatic digestion, cartilage pieces were pretreated with 0.25% trypsin/1mM EDTA for 45 minutes at 37°C and then incubated with 0.15% type II collagenase (Worthington Biochemical Corporation, Lakewood, NJ, USA) in Dulbecco’s modified Eagle’s medium (DMEM, Sigma Aldrich) overnight at 37°C with shaking [51]. HACs were seeded in plastic dishes and cultured in DMEM supplemented with 10% FBS, 2mM L-glutamine, antibiotics (100U/mL penicillin, 100 mg/mL streptomycin) and amphotericin B (0.25 mg/mL) at 37°C in a humidified atmosphere with 5% CO_2_. First-passage cultured chondrocytes were used for all experiments. For proliferation studies, HACs were seeded at density of 5x10^3^ cells/cm^2^ into 24-well plates. After over-night serum starvation, HACs were cultured in DMEM supplemented with 10% FBS. Proliferation was assessed after 1, 2, 4 and 6 days and expressed as ratio between counted (T1, T2, T3, T4) and seeded (baseline, T0) cells calculated by using a hemocytometer. Data were expressed as the average of three independent experiments performed in triplicate.

### Clusterin siRNA and cell viability

The following CLU siRNA (siCLU) sequences were purchased from Sigma Aldrich (St. Louis, MO, USA): sense 5’-GGAUGAAGGACCAGUGUGAdTdT-3’, antisense 5’-UCACACUGGUCCUUCAUCCdTdT-3’. In parallel, cells transfected with nonspecific siRNA (scramble) (sense 5’-UUCUCCGAACGUGUCACGUdTdT-3’, antisense 5’-ACGUGACACGUUCGGAGAAdTdT-3’) were used as control [52]. Lipofectamine 2000 reagent was used to transfect OA-HACs following the manufacturer’s suggested protocol (Invitrogen, Thermo Fisher Scientific, Massachusetts, USA) and 33nM siRNA/well was used as final working concentration. The 3-(4,5 dimethylthiazol-2-yl)-2,5 diphenyltetrazolium bromide assay (MTT; Sigma-Aldrich) was performed to test cell viability. For transient transfection, OA-HACs were seeded in 96-well cell culture plates and cell viability was tested after 24, 48 and 72 hours of incubation. All results obtained from CLU silencing were expressed as the average of three independent experiments performed in triplicate.

### Immunocytochemistry

HACs were seeded in 8 wells/chamber slides (cultured for 24 hours) or directly attached to glass slides by cytospin and fixed in 4% paraformaldehyde for 5 minutes at 4°C. After permeabilization in TBS-0.1% Tween 20 for 5min at RT, slides were incubated with rabbit polyclonal antibody anti-Ki67 (Novus Biologicals, CO, USA, 1:100, 1h at RT), goat polyclonal anti-CLU β (Santa Cruz Biotechnology, 1:50, O/N at +4°C), mouse monoclonal anti-p21 (R&D System Inc., USA, 8μg/ml, 1h at RT), rabbit polyclonal anti-cleaved caspase-3 (Cell Signaling Technology, Inc, MA, USA, 1:500, 1h at RT) and goat polyclonal anti-TNFα (Santa Cruz Biotechnology, 1:100, 2h at RT), with positive and negative controls. Diaminobenzidine was used as chromogen and Mayer’s haematoxylin as nuclear counterstain. Ki67, CLU, p21, cleaved caspase-3 and TNFα immunostainings were evaluated and expressed as percentage of positive cells in at least ten randomly selected fields at 400X magnification.

### RT-PCR and real-time PCR analysis

Total RNA extraction from cultured HACs of individual subjects (n=10 fractured and n=10 OA patients) and from cultured OA-HACs after 48 hours of CLU silencing (see “Clusterin siRNA and cell viability” paragraph) was performed using the TRIZOL reagent according to the manufacturer’s instructions (Invitrogen, Carlsbad, CA). Reverse-transcriptase polymerase chain reaction (RT-PCR) and real-time polymerase chain reaction (Real-time PCR) were performed using Platinum® Taq DNA Polymerase (RT-PCR, Invitrogen) and iQ^TM^ SYBR® Green Supermix (Real-time PCR, Bio-Rad, CA, USA) in an iQ5 Multicolor Real-time PCR Detection System (Bio-Rad). Specific primers are listed in Supplementary Table 3. For RT-PCR, results were normalized to the reference gene glyceraldehyde-3-phosphate dehydrogenase (GAPDH). Positive controls: NOX1 and NOX5, Caco-2; NOX2, phagocytes; NOX3, MCF-7; NOX4, Huvec [53]. For Real-time PCR, β-actin, β2-microglobulin and GAPDH were used as reference genes for normalization and gene expression calculated as fold change by 2^-ΔΔCT^ in triplicate experiments [54].

### cDNA plate array

Gene expression analysis was performed using Signosis array (Signosis, Inc. Santa Clara, CA, USA). Briefly, total RNA was extracted from OA-HACs after 48 hours of CLU silencing (see “Clusterin siRNA and cell viability” paragraph) and reverse-transcribed into cDNA in the presence of biotin-dUTP. A profile of 24 genes belonging to NFkB and Stat-3 signaling pathways (cDNA plate array) was analyzed and values expressed as relative light units (RLUs) according to the manufacturer’s instructions. Normalization was performed to the internal reference gene GAPDH. Reported data derived from three independent silencing experiments.

### Western blot analysis

Total cell lysates were extracted from HACs of individual subjects (n=10 fractured and n=10 OA patients) and from OA-HACs after 72 hours of CLU silencing (see “Clusterin siRNA and cell viability” paragraph) using lysis buffer containing phosphatase and protease inhibitors amd proteins quantified by Bradford assay. After separation by gradient sodium dodecyl sulfate-polyacrylamide gel electrophoresis, proteins were blotted to nitrocellulose transfer membranes and incubated with rabbit monoclonal anti-CLU (Cell Signaling Technology; 1:150, O/N), rabbit polyclonal anti-MMP13 antibody (Abcam; 1:500, O/N) and anti-NOX4 (Santa Cruz Biotechnology, USA; 1:200, O/N) and rabbit monoclonal anti-collagen X (Abcam; 1:1000, O/N). Normalization was performed using mouse monoclonal anti-GAPDH (Thermo Fisher Scientific; 1:2000, O/N).

### Detection of intracellular reactive oxygen species

ROS levels were measured by 5-(and-6)-chloromethyl-2’,7’-dichlorodihydrofluorescein diacetate, acetyl ester (CM-H2DCFDA) fluorescence method (Molecular Probes, Inc., Eugene, OR, USA). Briefly, after 12, 24 and 48 hours of siRNA transfection, HACs were incubated with 10μM of CM-H2DCFDA for 30 min at 37°C. The green fluorescence emission, indicative of the oxidation state, was visualized and photographed using Nikon Digital Camera DXM1200F connected to Nikon ACT-1 camera controller software. ROS level measurement was performed by analyzing at least 10,000 cells using a flow cytometer (Beckman Coulter, Brea, Calif., USA). Results were expressed as mean of three different experiments.

### Statistical analysis

Anonymized data were entered into a dataset. Statistical association tests were performed for cases and controls. For all analyses, *P* value < 0.05 was considered significant. For categorical variables (Alcian blue-PAS and secreted CLU evaluation), the Mann-Whitney’s U-test was performed. For quantitative variables, differences between two groups were analyzed by Student’s T-test. Results were presented as mean ± SEM (standard error of mean). All quantitative data sets presented here passed the normality tests. Homogeneous variance of the input data was confirmed using an F-test. Statistical analyses were performed with SPSS software (IBM, SPSS Statistics, US. version 23).

## Supplementary Material

Supplementary Tables
