# Supplementary material for: Clusterin exerts a cytoprotective and antioxidant effect in human osteoarthritic cartilage
Source: Aging (Albany NY). 2020 Jun 9;12(11):10129–46. doi: 10.18632/aging.103310 (PMC7346069; doi:10.18632/aging.103310)
Supplement: Supplementary Tables [file aging-12-103310-s001..pdf]

SUPPLEMENTARY TABLES

Supplementary Table 1. Clinical-pathological features and immunohistochemical results of fractured patients.

Patients	Group	Sex	Age	T-score	Alcian blue staining (AU)	% CLU^+ chondrocytes	CLU matrix staining (AU)	% $ACH4^+$ chondrocytes	% $IL6^+$ chondrocytes
1	Fractured	M	74	-3.3	3.0	8.0	1.0	1.1	1.1
2	Fractured	M	87	-3.3	3.0	11.0	2.0	11.1	2.0
3	Fractured	M	69	-2.2	2.5	4.2	1.0	9.2	5.6
4	Fractured	M	82	-2.4	1.0	18.3	1.0	0.0	8.4
5	Fractured	M	92	-2.9	2.0	6.0	0.5	0.0	3.0
6	Fractured	M	78	-2.5	1.0	19.1	1.5	10.0	0.0
7	Fractured	M	77	-1.9	3.0	8.4	0.5	14.0	0.0
8	Fractured	M	80	-1.5	3.0	2.8	0.0	8.5	0.0
9	Fractured	M	69	-2.2	1.5	15.6	0.5	13.0	1.1
10	Fractured	M	77	-2.1	1.5	9.3	1.0	5.1	1.2
11	Fractured	M	63	-2.4	1.5	26.5	2.5	4.3	0.9
12	Fractured	M	88	-3.1	2.5	14.5	1.0	0.0	11.5
13	Fractured	F	69	-1.7	3.0	4.0	0.5	12.4	0.5
14	Fractured	F	64	-0.9	2.5	8.9	1.0	3.0	1.5
15	Fractured	F	80	-3.5	1.0	17.6	1.5	2.2	0.6
16	Fractured	F	72	-0.7	3.0	9.7	1.0	5.4	16.6
17	Fractured	F	71	-2.6	2.5	28.1	3.0	2.5	0.0
18	Fractured	F	61	-3.0	2.0	13.5	1.0	1.0	9.5
19	Fractured	F	74	-3.7	1.5	11.9	1.0	6.4	1.0
20	Fractured	F	82	-3.2	3.0	8.4	0.5	10.3	4.5
21	Fractured	F	78	-2.8	2.0	3.3	0.0	8.9	4.9
22	Fractured	F	86	-2.2	2.5	4.2	0.5	0.0	1.4
23	Fractured	F	96	-3.7	1.5	16.7	1.5	9.8	2.2
24	Fractured	F	89	-2.2	3.0	9.6	1.0	0.0	2.1
25	Fractured	F	86	-0.8	1.0	24.6	2.0	15.2	0.0
26	Fractured	F	86	-2.7	1.5	5.6	0.5	1.5	1.6
27	Fractured	F	82	-1.7	3.0	7.8	1.0	0.0	0.6
28	Fractured	F	79	-2.7	3.0	9.0	1.0	0.0	1.0
29	Fractured	F	93	-3.5	2.0	10.5	1.0	0.0	2.2
30	Fractured	F	80	-2.8	1.0	19.7	1.0	30.8	0.0

Abbreviation: AU, arbitrary units.

Supplementary Table 2. Clinical-pathological features and immunohistochemical results of osteoarthritis patients.

Patients	Group	Sex	Age	K/L	HHS	T-score	Alcian blue staining (AU)	% CLU^+ chondrocytes	CLU matrix staining (AU)	% $ACH4^+$ chondrocytes	% $IL6^+$ chondrocytes
1	OA	M	66	3	61	1,5	0,0	56,9	1,0	37,8	60,0
2	OA	M	79	4	52	-1,2	0,0	46,7	1,5	62,6	76,3
3	OA	M	74	4	39	2,3	0,0	30,8	1,5	34,4	19,7
4	OA	M	74	3	60	0,2	1,5	58,3	3,0	85,0	52,0
5	OA	M	73	4	45	2,0	0,0	59,0	2,5	12,3	82,5
6	OA	M	80	4	47	-0,7	1,0	51,2	2,0	10,2	82,1
7	OA	M	79	3	61	0,3	0,0	82,3	3,0	42,1	8,1
8	OA	M	67	3	66	1,4	0,0	33,9	1,0	14,7	67,9
9	OA	M	69	3	53	-1,4	2,0	54,8	2,0	26,4	23,2
10	OA	M	67	4	53	1,0	1,0	41,2	1,5	65,9	76,5
11	OA	M	78	4	43	0,4	0,5	53,6	1,5	63,6	81,0
12	OA	M	81	3	42	-1,5	0,0	38,5	1,5	80,0	20,1
13	OA	M	79	4	20	-1,7	1,5	41,6	2,0	22,5	41,5
14	OA	M	74	3	43	1,5	0,0	26,0	1,0	18,4	54,0
15	OA	F	67	3	58	-0,9	0,5	64,4	2,5	5,1	53,4
16	OA	F	78	4	54	3,3	0,5	51,3	1,5	50,1	73,1
17	OA	F	80	3	44	-1,4	0,0	90,0	3,0	43,7	23,2
18	OA	F	75	3	39	-0,5	0,0	39,5	2,0	34,3	88,7
19	OA	F	77	4	33	0,0	2,0	59,5	1,5	78,0	7,5
20	OA	F	76	4	22	0,4	0,0	37,0	1,0	3,2	78,2
21	OA	F	80	4	50	-1,4	0,5	52,4	2,0	34,8	68,7
22	OA	F	67	3	58	-1,6	1,0	37,6	1,5	39,0	95,0
23	OA	F	87	3	40	-0,9	1,5	38,6	1,0	18,6	9,9
24	OA	F	66	4	14	-2,2	0,0	52,3	1,5	78,6	33,3
25	OA	F	85	4	69	-0,9	0,0	45,5	1,5	65,9	47,0
26	OA	F	75	3	36	-0,7	0,0	61,9	2,0	22,8	90,0
27	OA	F	69	3	41	-1,4	2,0	60,6	3,0	4,1	12,0
28	OA	F	73	3	46	-1,1	0,0	40,1	1,5	89,0	50,1
29	OA	F	80	4	18	-2,0	0,5	14,2	0,5	64,3	59,9
30	OA	F	76	3	34	-1,4	0,0	36,3	1,5	6,1	71,0

Abbreviations: K/L, Kellgren-Lawrence Score; HHS, Harris Hip Score; AU, arbitrary units.

Supplementary Table 3. Primers used for RT-PCR or real-time PCR.

Gene	Primer sequence	Accession number	Tann (°C)
**CLU (RT-PCR)**	Sense: 5’-GTGCAATGAGACCATGATGG- 3’<br>Antisense: 5’-CAGGTAGTGGTAGGTATCCT- 3’	NM_002427.3	55
**CLU (Real-Time)**	Sense: 5’-ACAGGGTGCCGCTGACC- 3’<br>Antisense: 5’-CAGCAGAGTCTTCATCATGCC- 3’	NM_001831.3	60
**NOX1**	Sense: 5’-CTTGCCTCCATTCTCTCCAG- 3’<br>Antisense: 5’-CACTCCAGTGAGACCAGCAA- 3’	NM_007052.4	56
**NOX2**	Sense: 5’-CCAGTGAAGATGTGTTCAGCT- 3’<br>Antisense: 5’-GCACAGCCAGTAGAAGTAGAT- 3’	NM_000397.3	60
**NOX3**	Sense: 5’-GCCCAACTGGAACAATGAGT- 3’<br>Antisense: 5’-ATGAACACCTCTGGGGTCAG- 3’	NM_015718.2	60
**NOX4**	Sense: 5’-CTCAGCGGAATCAATCAGCTGTG- 3’<br>Antisense: 5’-AGAGGAACACGACAATCAGCCTTAG- 3’	NM_016931.4	58
**NOX5**	Sense: 5’-GCTGTCGAGGAGTGTGACAA- 3’<br>Antisense: 5’-GCTCAGAGGCAAAGATCCTG- 3’	NM_024505.3	60
**COL2A1**	Sense: 5’-CAACACTGCCAACGTCCAGAT- 3’<br>Antisense: 5’-CTGCTTCGTCCAGATAGGCAAT- 3’	NM_001844.4	60
**ACAN**	Sense: 5’-CATTCACCAGTGAGGACCTCGT- 3’<br>Antisense: 5’-TCACACTGCTCATAGCCTGCTTC- 3’	NM_001135.3	60
**COL10A1**	Sense: 5’-GCACGCAGAATCCATCTGAGAATA- 3’<br>Antisense: 5’-GACCAGGAGTACCTTGCTCTC- 3’	NM_000493.3	60
**MMP13**	Sense: 5’-ATGCATCCAGGGGTCCTGGC- 3’<br>Antisense: 5’-TGCTGCATTCTCCTTCAGGA- 3’	NM_001135.3	60
**GAPDH**	Sense: 5’-ACGGATTTGGTCGTATTGG- 3’<br>Antisense: 5’-GATTTTGGAGGGATCTCGC- 3’	NM_002046	60
**β-actin**	Sense: 5’-CTGGAACGGTGAAGGTGACA- 3’<br>Antisense: 5’-AAGGGACTTCCTGTAACAATGCA- 3’	NM_001101.3	60
**β2-microglobulin**	Sense: 5’-GATTCAGGTTTACTCACGTC- 3’<br>Antisense: 5’-GTTCACACGGCAGGCATACT- 3’	NM_004048	60
